# Investigations of reported plasma amyloid b peptides and total tau protein assayed with immunomagnetic reduction in cognitively normal controls and patients with amnestic mild cognitive impairment and Alzheimer’s disease dementia

**DOI:** 10.1002/alz.090058

**Published:** 2025-01-09

**Authors:** Charles S.Y. Yang

**Affiliations:** ^1^ MagQu Co., Ltd., Taiwan, New Taipei City Taiwan

## Abstract

**Background:**

Since 2012, many groups have been using immunomagnetic reduction (IMR) for assaying plasma amyloid β 1‐40 (Aβ_1‐40_) peptide, Aβ_1‐42_ peptide and total tau protein (T‐Tau) in cognitively normal controls (NC) and patients with amnestic mild cognitive impairment (aMCI) and Alzheimer’s disease dementia (ADD). Tremendous results have been independently reported. In this work, we summarize the reported levels of plasma Aβ_1‐40_, Aβ_1‐42_ and T‐Tau to investigate the consistence among studies.

**Method:**

Results of thirty studies were analyzed, including twenty‐five studies published in papers, two studies presented at conferences and three unpublished studies.

**Result:**

The coefficient of variation (CV) in reported mean values of biomarker concentrations among studies are 8.1% for Aβ_1‐40_, 4.1% for Aβ_1‐42_ and 19.4% for T‐Tau in NC, 10.7% for Aβ_1‐40_, 8.4% for Aβ_1‐42_ and 15.0% for T‐Tau in aMCI, 10.6% for Aβ_1‐40_, 19.1% for Aβ_1‐42_ and 28.7% for T‐Tau in ADD. In the thirty studies, there are 1189 subjects of NC, 543 aMCI patients and 853 ADD patients. The averaged level of plasma Aβ_1‐40_ of subjects was found to significantly decrease from NC (59.72 pg/ml) to aMCI (45.92 pg/ml, p < 0.0001), which showed non‐significant difference from ADD (48.34 pg/ml, p > 0.05). The averaged level of plasma Aβ_1‐42_ significantly increased from NC (15.72 pg/ml) to aMCI (17.66 pg/ml, p < 0.0001), which was not significantly different from ADD (19.39 pg/ml, p > 0.05). However, the averaged level of plasma T‐Tau significantly increased from NC (17.92 pg/ml) to aMCI (28.26 pg/ml, p < 0.0001), further significantly increased to ADD (35.51 pg/ml, p < 0.001). The summarized results reveal important impacts to clinical utilities of plasma biomarkers in AD.

**Conclusion:**

The measured concentrations of plasma Aβ_1‐40_, Aβ_1‐42_ and T‐Tau with IMR are consistent and reliable among studies. Furthermore, the plasma Aβ_1‐40_, Aβ_1‐42_ and T‐Tau levels are able to discriminate NC from patients with aMCI and ADD. Plasma T‐Tau levels are disease‐severity dependent.

